# The ability of microRNAs to regulate the immune response in ischemia/reperfusion inflammatory pathways

**DOI:** 10.1038/s41435-024-00283-6

**Published:** 2024-06-22

**Authors:** Peter Artimovič, Ivana Špaková, Ema Macejková, Timea Pribulová, Miroslava Rabajdová, Mária Mareková, Martina Zavacká

**Affiliations:** 1 Department of Medical and Clinical Biochemistry, Pavol Jozef Šafárik University in Košice, Faculty of Medicine, Košice, Slovakia; 2 Department of Vascular Surgery, Pavol Jozef Šafárik University in Košice, Faculty of Medicine, Košice, Slovakia

**Keywords:** Gene regulation, Chronic inflammation, Inflammasome, NF-kappaB, Acute inflammation

## Abstract

MicroRNAs play a crucial role in regulating the immune responses induced by ischemia/reperfusion injury. Through their ability to modulate gene expression, microRNAs adjust immune responses by targeting specific genes and signaling pathways. This review focuses on the impact of microRNAs on the inflammatory pathways triggered during ischemia/reperfusion injury and highlights their ability to modulate inflammation, playing a critical role in the pathophysiology of ischemia/reperfusion injury. Dysregulated expression of microRNAs contributes to the pathogenesis of ischemia/reperfusion injury, therefore targeting specific microRNAs offers an opportunity to restore immune homeostasis and improve patient outcomes. Understanding the complex network of immunoregulatory microRNAs could provide novel therapeutic interventions aimed at attenuating excessive inflammation and preserving tissue integrity.

## Introduction

Temporary interruption of blood flow to a tissue or organ by trauma, vascular obstruction, or different flow-limiting diseases results in hypoxia and ischemic injury of the tissue [1]. Ischemia contributes to various pathologies that clinicians face daily including stroke, myocardial infarction, peripheral vasculopathy, organ transplantation, trauma, and surgeries involving temporary vascular occlusion [2, 3]. The main factor contributing to the augmentation of ischemic injury is the subsequent restoration of blood flow. While reperfusion is essential to rescue the affected tissue, paradoxically it leads to a robust inflammatory response, contributing to tissue damage, and dysfunction [4]. Local inflammation, accompanied by cell death during reperfusion, is known as ischemia/reperfusion (I/R) injury [5].

The microenvironment of I/R injury is composed of complex interactions between the immune, muscular, and vascular systems [6]. The immune response plays a crucial role in I/R injury as it involves activating various immune cells, the release of inflammatory mediators, and recruiting immune effector cells [7]. Among the regulators of immune responses, microRNAs (miRNAs) have emerged as key players in modulating intricate inflammatory pathways, including the NLR family pyrin domain containing 3 (NLRP3) inflammasome, Toll-like receptor (TLR) signaling, cytokine production, and immune cell activation. These small non-coding RNAs (ncRNAs) have the ability to target specific areas of messenger RNAs (mRNAs) and regulate their stability and translation to influence the outcome of I/R injury [8].

## The interplay of miRNAs and immune responses in I/R

Deprivation of oxygen and nutrients during ischemia may lead to cell injury and death. Low levels of oxygen trigger a metabolic shift towards anaerobic metabolism resulting in the accumulation of various metabolites and cellular acidification [7]. Production of ATP is decreased due to low oxygen levels and disrupted oxidative phosphorylation in mitochondria, causing cellular levels of ATP to rapidly fall, promoting dysfunction of ATP-dependent membrane pumps, and leading to oncotic cell death [9]. Limiting the ischemic time is crucial for maintaining the vitality of tissues after reperfusion. Cells in hypoxia activate pro-angiogenic mechanisms by stabilizing HIF-1α to promote angiogenesis and restore tissue blood flow [10]. However, reperfusion following ischemia is also detrimental due to the increased production of reactive oxygen species (ROS) which damage cellular components promoting apoptotic or necrotic pathways [11].

Damaged tissues release damage-associated molecular patterns (DAMPs), inducing an immune response and causing local inflammation [12]. Nuclear protein high mobility group box 1 (HMGB1), extracellular ATP, histones, or mitochondrial DNA released from necrotic cells stimulate the NLRP3 inflammasome which in turn activates caspase-1 to promote interleukin IL-1β and IL-18 production during pyroptosis, an inflammatory form of programmed cell death [13]. Intracellular signalization by DAMPs is based on their interaction with pattern-recognition receptors (PRRs) expressed by immune cells, however, PRRs can also be expressed by endothelial and epithelial cells [14]. Pattern-recognition receptors are a heterogeneous group of receptors including TLRs, NOD-like receptors (NLRs), C-type lectin receptors (CLRs), RIG-I-like receptors (RLRs), receptors for advanced glycation end-products (RAGE), or extracellular ATP sensors with P2X motif and they can be expressed in membrane-bound, intracellular, or secreted soluble forms [15]. The effector molecule of PRRs signaling is the nuclear factor kappa-B (NF-κB), the main transcription factor of inflammatory genes [14]. Neutrophils may respond to inflammatory stimuli by undergoing NETosis, a unique neutrophile-specific form of cell death. Immunogenic extracellular nets formed during NETosis consist of DNA, histones, and various enzymes, such as myeloperoxidase or neutrophil elastase [16]. Increased levels of pro-inflammatory cytokines prime endothelium for activation by increasing the expression of vascular cell adhesion molecule 1 (VCAM-1, CD106) and intracellular adhesion molecule 1 (ICAM-1, CD54), thus enhancing leukocyte adhesion and transmigration [17]. The pro-inflammatory microenvironment promotes M1 macrophage polarization and neutrophil activation, further promoting endothelial dysfunction [18]. As a result, the resolution of inflammation is delayed, forming a vicious cycle of ischemia/reperfusion-induced immune responses.

The role of ncRNAs in immune responses is widely studied in the recent decade. The most intensively studied ncRNAs are microRNAs (miRNAs/miRs) which have about 18–24 nucleotides [19]. Studies have shown that more than 60% of human coding genes are regulated by miRNAs, therefore their regulatory role may significantly contribute to the onset of diseases [8]. MiRNAs mainly regulate gene expression at the post-transcriptional level via binding to the 3´- untranslated region (UTR) of mRNA, however, miRNAs can also interact with 5´-UTR regions, coding sequences, or promotors [20]. Their ability to post-transcriptionally regulate gene expression shows their diagnostic potential, since their dysregulation precedes detectable changes in protein levels. The expression of miRNAs is cell- and tissue-specific, therefore their interactome may vary accordingly [21].

It is very difficult to sort miRNAs into distinct categories since many of them regulate various molecular pathways simultaneously [8]. However, grouping miRNAs with similar effects and studying the overlap of these groups may help researchers better understand the complex miRNA network in various pathologies.

For instance, inflammatory miRNAs represent a group of miRNAs that either directly regulate immune processes or their expression level is changed during inflammation [22]. Anti-inflammatory miRNAs attenuate inflammatory processes and directly target important mediators of immune responses, such as myeloid differentiation primary response 88 (MyD88) or interleukin-1 receptor-associated kinase (IRAK), and tumor necrosis factor (TNF) receptor-associated factor (TRAF) proteins [23]. Furthermore, miRNAs can directly inhibit the NLPR3 inflammasome, leading to a significant reduction in the production of proinflammatory cytokines [24]. In contrast, pro-inflammatory miRNAs promote inflammation by downregulating negative regulators of inflammatory pathways, such as sirtuins [25].

Reperfusion of tissues is also modulated by angiogenesis as was mentioned. Most miRNAs targeting SIRT1 are pro-inflammatory and anti-angiogenic, based on the function of SIRT1 in cell biology [26]. Most notably, miRNAs that were presented to play a role in tumorigenesis were also associated with increased angiogenesis and attenuated inflammation [27]. In addition, hypoxia stimulates angiogenesis through the hypoxia-inducible factor 1α/vascular endothelial growth factor (HIF-1α/VEGF) pathway [28]. The expression pattern of miRNAs responds to hypoxia by upregulation of pro-angiogenic and downregulation of anti-angiogenic miRNAs to increase the level of oxygen and nutrients in the microenvironment [29].

## MiRNA-regulated inflammatory pathways activated during I/R

Cells respond to oxidative stress by triggering repair mechanisms or undergoing apoptosis to prevent malignant transformation [7]. Exudation of cell contents during oncotic cell death induced by I/R activates the immune system through various inflammatory pathways including NLRP3, TLR, TNF or mTOR signaling [12].

### Activation of mTOR pathway in I/R

Kinase cascades in inflammatory pathways may converge into the mammalian target of rapamycin (mTOR) signaling pathway mainly through phosphoinositide 3-kinase/protein kinase B (PI3K/Akt) activation [30]. The mTOR is a serine/threonine-specific protein kinase that influences cell growth, proliferation, survival, protein synthesis, ribosome biogenesis, autophagy, metabolism, and immune response. It exists in two complexes, mTOR complex 1 (mTORC1) and mTOR complex 2 (mTORC2) both with different functions [31]. In the context of inflammation, mTOR has been shown to have anti- and pro-inflammatory effects, depending on the cell type, tissue environment, and overall combination of cellular signals. Activity of mTORC1 can induce the NF-κB and stimulate the production of IL-1β, IL-6, and TNF-α to promote inflammation [32]. The mTORC1 also enhances the translation of inflammatory proteins by regulating ribosomal protein synthesis [33]. The role of mTORC2 in inflammation is not entirely understood in contrast to the extensively studied mTORC1 [34]. Studies suggested its role in polarization of macrophages towards the M2 phenotype, resulting in the production of anti-inflammatory cytokines [32]. However, mTORC2 can indirectly influence mTORC1 signaling by positively regulating Akt which activates the mTORC1 [35]. Cellular stress during ischemic or reperfusion phase of the I/R injury increases mTORC1 signaling which exacerbates inflammatory damage to tissues [36]. Apart from the activation of inflammatory transcription factors, mTORC1 impairs mitochondrial function and increases ROS production which further contributes to the destruction of cells [37].

**Fig. 1:**
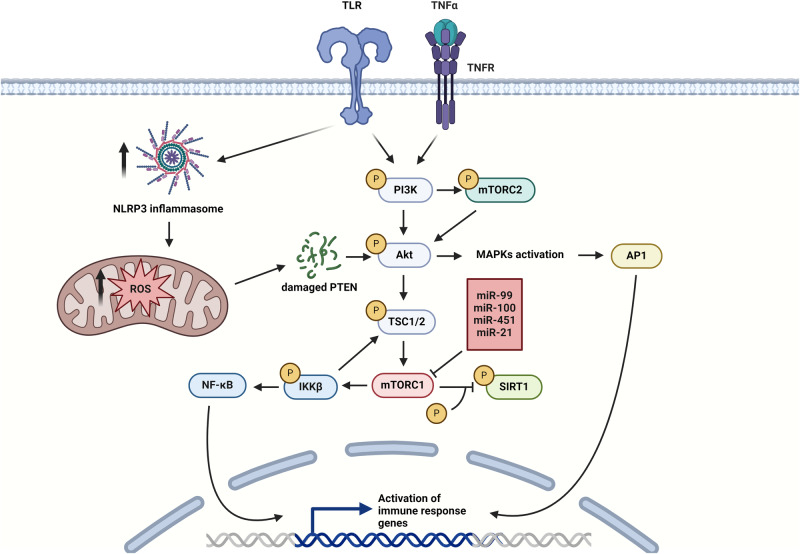
Activation of mTOR pathway via TLR, TNF and NLRP3 signaling. Initiation of kinase cascades in TLR and TNF signaling leads to phosphorylation of PI3K and activation of PI3K/Akt/mTOR pathway to promote the expression of inflammatory genes. Induction of NLRP3 by these inflammatory pathways indirectly modulates mTOR activity by upregulating production of ROS to damage PTEN – an inhibitor of Akt kinase. Activity of mTORC1 is negatively regulated by several miRNAs repressing its translation, thus dampening the inflammation. (Created with BioRender.com).

MicroRNAs can also regulate and target mTOR pathway to modulate inflammation (Fig. 1). Recent findings indicate that miR-99 specifically targets mTOR mRNA in naive CD4^+^ T cells, leading to an increased prevalence of regulatory T cells (Tregs). Augmentation of miR-99 expression may ameliorate the extensive inflammatory response observed during tissue reperfusion [38]. Conversely, miR-100, which similarly modulates mTOR, has been observed to diminish Treg populations, potentially exacerbating ischemia/reperfusion (I/R) injury [39]. Another miRNA regulating the mTOR activity is miR-451, which increases its activity to promote inflammation, as shown in tumor-infiltrated T cells [40]. Apart from CD4^+^ T cells, the regulatory role of microRNAs extends to CD8^+^ T cells, with miR-155, for example, enhancing cytotoxic responses and adversely affecting tissue rescue outcomes [41]. Study on murine macrophages has shown anti-inflammatory effect of miR-21 which indirectly promotes mTOR signaling by targeting phosphatase and tensin homolog (PTEN) to enhance M2 polarization in macrophages [42]. Few other miRNAs also target the components of the mTORC1 complex including miR-1224-5 targeting the polo-like kinase 1 (PLK1) to inhibit activation of PI3K/Akt/mTOR pathway or miR-199a which targets multiple components of the mTOR pathway and its downstream effectors in human cardiomyocytes [43, 44].

### The NLRP3 inflammasome pathway in I/R

Restoration of blood flow in ischemic tissues may activate the pyroptotic pathway including the activation of NLRP3 inflammasome [45]. This multiprotein complex consists of cytosolic NLRs capable of recognizing DAMPs or danger signals induced by pathogen-associated molecular patterns (PAMPs) [46]. Expression of the inflammasome is positively regulated by NF-κB [45]. Transcribed mRNA of NLRP3 is the target of anti-inflammatory miR-223 [47]. Interestingly, the expression of this miRNA can be increased by the action of NF-κB suggesting a regulatory feedback loop of inflammation [48]. On the other hand, miR-9 exerts an indirect downregulation of the NLRP3 inflammasome pathway in cardiomyocytes through its targeting of ELAV-like protein 1 (ELAVL1, HuR), a critical regulator involved in the stabilization of NLRP3 transcript, promoting pyroptosis [49]. Indirect regulation of NLRP3 expression can also occur through the action of miR-30c-5p, as observed in a study conducted on human aortic endothelial cells (HAECs) in the context of atherosclerosis [50]. Results of this study have shown that suppression of forkhead box O3 (FOXO3) mediated by miR-30c-5p protects HAECs from damage induced by oxidized LDL and causes decreased levels of NLRP3, replication-associated protein (AC1), IL-18, and IL-1β [50]. In addition, activation of mTORC1 has been demonstrated to increase the levels of NLRP3 through elevated ROS generation. Therefore, inhibition of mTORC1 by rapamycin suppresses NLRP3 inflammasome activation as well [37].

**Fig. 2:**
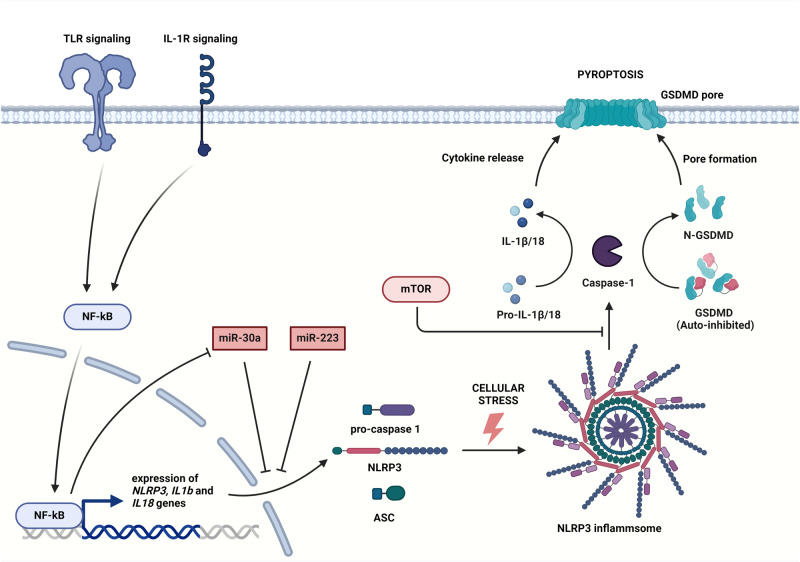
Inflammation-induced activation of NLRP3 leads to pyroptosis. Induction of TLR or IL-1R signaling promotes activity of NF-κB to activate the expression of *NLRP3*, *IL1b* and *IL18* genes. Cellular stress facilitates the assembly of NLRP3 inflammasome and maturation of caspase-1 resulting in a formation of GSDMD pore by cleaved GSDMD and release of active pro-inflammatory cytokines that are hallmarks of pyroptosis. The NLRP3 inflammasome pathway may be negatively regulated by miRNAs and mTOR as well. (Created with BioRender.com).

Maturation of NLRP3 inflammasome consists of signal transduction by DAMP/PAMP-receptor ligation, the association of NLRP3 with apoptosis-associated speck-like protein (ASC) and the last step is the recruitment of procaspase-1 through caspase activation and recruitment domain (CARD) interaction [45]. Procaspase-1 is a zymogen that is activated by auto-cleavage after associating with NLRP3, producing mature caspase-1 to cleave pro-interleukin-1β and pro-interleukin-18 to promote sterile inflammation and processing of the gasdermin D (GSDMD) [13]. Interestingly, study of Schmitz *et al*. (2008) has reported that processing of caspase-1 is negatively regulated by mTOR [51]. Units of GSDMD form a pore in the cell membrane (Fig. 2) resulting in lysis and release of inflammatory cytokines and thus pyroptosis [52]. The maturation of GSDMD is also crucial in NETosis. Production of ROS can trigger myeloperoxidase to release neutrophil elastase from azurophilic granules, cleave GSDMD during lytic NETosis, and promote inflammation [53].

Murine models of myocardium infarction and cerebral ischemia are frequently used to study the activation of NLRP3 inflammasome related to ischemic insults [52, 54]. Occlusion of arteries due to atherosclerotic plaques or formation of thrombi leads to hypoperfusion and loss of function [55]. MiR-223 targeting NLRP3 was reported to have a cardioprotective effect on myocardial inflammatory injury, during in vitro and in vivo experiments and its inhibition led to an increased formation of foam cells and inflammatory cytokine production in the atherosclerotic mice models. Additionally, miR-223 is considered a myeloid-lineage-specific miRNA and is especially important in the negative regulation of neutrophil and macrophage activity [24].

During inflammation, NF-κB downregulates miR-30a, a direct inhibitor of NLRP3 expression (Fig. 2) in macrophages, while simultaneously upregulating NLRP3 expression [56]. An important finding in the context of I/R injury is that miR-30a ameliorates oxidative stress via activation of nuclear factor erythroid 2-related factor 2/antioxidant responsive element (Nrf2/ARE) signaling pathway, however, this effect was studied in synovial fibroblasts of rheumatoid arthritis model and requires further investigation in hypoxia/reoxygenation cell culture models to investigate its therapeutic potential [57]. A study on a mouse model of hind limb ischemia has insinuated that inhibition of heme oxygenase 1 (HO-1) may alleviate tissue injury by increasing autolysosomal degradation of NLRP3 [50]. Similar results were discussed in models of cerebral ischemia where inhibition of NLRP3 inflammasome decreased neuronal death, M1 microglial polarization, and increased blood-brain barrier integrity [58]. Pharmacologically targeting the NLRP3 inflammasome pathway by silencing the expression of *NLRP3* and *GSDMD* genes, or by blocking caspase-1 downstream signaling, may provide a promising therapy to preserve endothelial integrity and alleviate inflammation in I/R injury [45].

### The role of TLR signaling in I/R

Toll-like receptors are essential components of innate immunity. In humans, ten types were identified so far [59]. Among them, six receptors (TLR1, TLR2, TLR4, TLR5, TLR6, and TLR10) are located on the cell surface and are recognized by exogenous and endogenous ligands and the other four receptors (TLR3, TLR7, TLR8, TLR9) are intracellular, located on endosomes and are generally activated by nucleic acids [60]. Homodimerization is typical for the TLR4 receptor, while TLR2 forms heterodimers with TLR1 and TLR6 receptors [61]. Based on the interaction of the intracellular domain of TLRs with adaptor molecules, the signaling cascade is divided into two types: MyD88-dependent pathway and TIR-domain-containing adapter-inducing interferon-β (TRIF)-dependent pathway [59]. The initiation of the signaling cascade may include the binding of miRNAs to TLRs such as the binding of let-7i to TLR4 or the binding of exosomal miRNAs to TLR7 and TLR8 receptors on immune cells. On the transcriptional level, let-7i downregulates TLR4 signaling in human cholangiocytes by directly inhibiting TLR4 expression [62].

Adaptor protein MyD88 recruits TIR domain-containing adaptor protein (TIRAP, Mal) to initiate TLR2/TLR4 signaling [60]. There are several miRNAs with an ability to target MyD88 in TLR signaling such as miR-155, miR-203-5p, miR-149-5p, and miR-124-5p, however, these studies have also found that aforementioned miRNAs, except miR-155, suppress the translation rather than cause mRNA degradation of MyD88 in mouse macrophages [63–66]. Complex formed from TIRAP and MyD88 recruits and activates IRAK4, resulting in the phosphorylation of IRAK1 that associates with TRAF6 [57]. The miR-146a acts as a negative regulator of TLR4 in macrophages by directly targeting IRAK1 and TRAF6, thereby inhibiting LDL accumulation and suppressing the inflammatory response [67, 68]. The importance of miR-146a is underscored by a study showing that insufficient degradation of TRAF6 can promote tissue damage by increasing NF-κB activity and promoting unregulated ubiquitination, leading to autophagy-related cell damage. This effect of dysregulated TRAF6 degradation was studied in HeLa and human embryonic kidney cells 293 (HEK293) under LPS-triggered inflammatory signaling, indicating that the potential for targeting TRAF6 in I/R injury shows great promise but requires further clarification [69, 70]. This miRNA also belongs to a group of early response genes and is highly induced after TLR stimulation in human monocytes [71]. Downregulation of IRAK1 can be compensated by IRAK2, also a target of miR-146a [72]. Several other miRNAs target IRAK1, such as miR-133-5p and miR-142-5p, and member of the IRAK family, IRAK4 is the target of miR-93-5p [73–75]. Phosphorylated TRAF6 dissociates from the receptor to form a complex with transforming growth factor-β-activated kinase 1 (TAK1), TAK1-binding protein 1 (TAB1), and TAK1-binding protein 2 (TAB2) [57]. Expression of TAB2 can be suppressed by the action of miR-155 resulting in the attenuation of the inflammatory signal [76]. Association of ubiquitin ligases ubiquitin-conjugating enzyme 13 (UBC13) and ubiquitin-conjugating enzyme E2-variant 1 (UEV1A) with the TAK1 promotes its activation which phosphorylates mitogen-activated protein kinase kinase 3 (MKK3), MKK6, and MKK7 to phosphorylate the IκB kinase (IKK) complex, activate p38 kinase and inhibitor of nuclear factor kappa-B kinase subunit beta (IKKβ) [77, 78]. Kinase p38 activates c-jun kinase (JNK), which translocates to the nucleus and activates the transcription factor complex activator protein 1 (AP1), promoting the expression of pro-inflammatory cytokines, leading to exacerbated tissue damage [78]. A transcriptomic study on smooth muscle cell-specific miRNAs and smooth muscle markers in patients with acontractile bladders has revealed that miR-199a can attenuate the inflammatory process by targeting apoptosis signal-regulating kinase 1 (ASK1), a key activator of the p38 signaling cascade [79]. The upregulation of miR-199a may serve as a beneficial intervention for patients suffering from I/R injury, due to its protective effects against inflammation-induced damage to muscle cells [44]. However, miR-199a also targets the IKKβ [79]. When activated by the IKK complex, IKKβ is ubiquitylated and degraded, releasing the functional transcription factor NF-κB, which promotes the inflammatory process. Therefore, upregulation of miR-199a may attenuate the AP1 pathway but promote NF-κB mediated inflammatory processes, suggesting simultaneous administration of different anti-inflammatory miRNAs in effectively managing the I/R injury [44, 79, 80]. Notably, IKK may additionally activate mTOR signaling via phosphorylation and inactivation of the inhibitory tuberous sclerosis 1 (TSC1)/TSC2 complex to initiate a mTORC1/NF-κB inflammatory cycle (Fig. 1) [81].

**Fig. 3:**
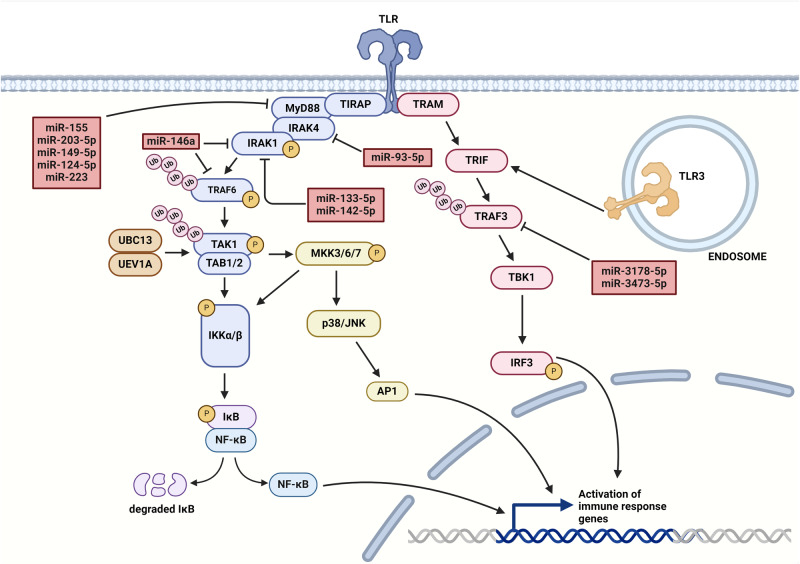
MiRNAs regulate various molecular pathways of TLR signaling. TLR pathway diverges into two signaling pathways mediated by MyD88 and TRAM proteins. The MyD88 pathway includes the activation of TIRAP/MyD88/IRAK4/IRAK1/TRAF6 signalosome leading to kinase cascades that promote the expression of immune response genes mediated by NF-κB and AP1 transcription factors. The TRAM pathway signals through TRAM/TRIF/TRAF3 signalosome to promote IRF3-mediated transcription of inflammatory genes. Ubiquitination of proteins serves as an activation mechanism in these pathways. Both pathways can be negatively regulated by several miRNAs to mitigate inflammation. (Created with BioRender.com).

Another TLR pathway is mediated by the TRIF adaptor protein [80]. The signal is transduced via the TRIF-related adaptor molecule (TRAM) associating with TRIF leading to the activation of TANK binding kinase 1 (TBK1) which phosphorylates and activates interferon-regulatory factor 3 (IRF3) [56]. TRIF-mediated activation of IRF3 signals through TRAF3 (Fig. 3), targeted by miR-3178-5p and miR-3473-5p as well [82, 83]. This pathway may also activate NF-κB by TRAF6 recruitment [80]. Most TLRs are mediated mainly through the MyD88-dependent pathway, except TLR3 which signalizes only via the TRIF-dependent pathway. In addition, TLR4 can also exert its action through the TRIF-dependent pathway [59]. The TLR signaling may also activate mTOR via MyD88-TRIF/PI3K/Akt pathway, although the mTOR can be also recruited to the MyD88 during assembly of IRAKs and TRAFs [84].

The release of nucleic acids during I/R injury stimulates TLR3/7/8/9 receptors to promote inflammatory response [85]. The study of murine renal I/R injury demonstrated that while pharmacological inhibition of TLR signaling had protective effects during the initial stage of injury, TLR knockout models only experienced a delay in ischemic injury without complete prevention [86]. This study suggests that the potential administration of miRNAs as pharmaceutical inhibitors targeting TLR signaling, such as those mentioned in this section, does not provide an absolute protection from I/R injury, since the TLR pathway is just one of many inflammatory pathways activated by I/R. Upregulated TLRs were shown to exacerbate I/R injury also in models of myocardial infarction and stroke, which supports their role as general propagators of inflammatory signal [87]. Regeneration of tissues after ischemia may be blocked by thrombosis induced by TLR4/NLRP3 pathway activation in platelets as was demonstrated in the murine model of hind limb ischemia [88]. Increased expression of miR-223 may inhibit the progression of atherosclerosis by blocking TLR4 signaling and activating the PI3K/Akt pathway [24].

### Implications of TNF signaling in I/R

**Fig. 4:**
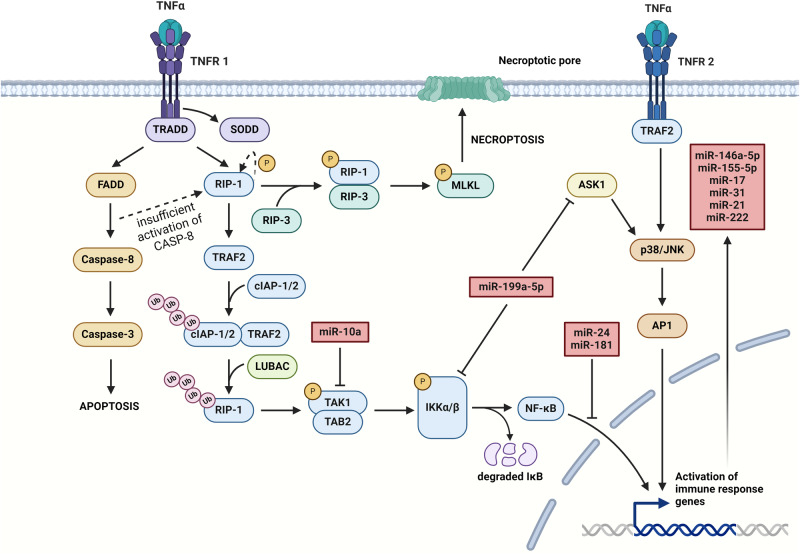
Molecular pathways of TNF signaling. Cytokine TNF-α can bind to two receptors that differ in structure and therefore their signaling. Most inflammatory processes are mediated by the main receptor – TNFR-1, however binding to TNFR-2 also promotes inflammation. Signaling by TNFR-1 includes recruitment of adaptor protein TRADD followed by the release of SODD and initiation of apoptotic, necroptotic or inflammatory signaling mediated by FADD, RIP-1/RIP-3 and RIP-1/TAK1/TAB2 respectively. Initial apoptotic pathway switches to necroptotic if the activation of caspase-8 is insufficient. Receptor TNFR-2 promotes the activation of AP1 to enhance the expression of immune response genes. Ubiquitination of proteins serves as an activation mechanism in these pathways. Several miRNAs can attenuate the inflammation via inhibition of important pro-inflammatory signaling molecules. In addition, few mostly anti-inflammatory miRNAs are upregulated by TNF signaling. (Created with BioRender.com).

Increased production of TNF-α during I/R injury leads to an extensive production of pro-inflammatory cytokines, promoting further tissue damage after reperfusion [54]. Most biological effects are related to TNF/TNF receptor 1 (TNFR-1) signal transduction even though it can also bind to the TNFR-2 receptor [89, 90]. A major step in TNF signaling pathway is the formation of a multiprotein complex near the TNFR-1 receptor that begins with the binding of the TNF trimer to this receptor and triggers the release of the inhibitory protein silencer of death domains (SODD) (Fig. 4) from the intracellular domain of the receptor [91]. On the other hand, TNFR-2 receptor contains only TRAF binding site and does not directly stimulate cell death [92]. The aggregated intracellular domain of TNFR is recognized by TNF receptor-associated death domain (TRADD) and may recruit receptor-interacting protein 1 (RIP-1) and TRAF2, along with the Fas-associated death domain (FADD) [93]. The TNF signaling consists of several inflammatory pathways explored further in this section [93].

The first TNF pathway involves FADD, following the recruitment of caspase-8 and initiation of caspase-mediated apoptosis [94]. Insufficient activation of caspase-8 may lead to the autophosphorylation of RIP-1 and form a heterodimer with RIP-3 leading to its autophosphorylation and producing a mature necrosome [93]. Activation of RIP-1/3 dependent necroptotic cell death is mediated by phosphorylation of mixed lineage kinase domain-like (MLKL) by RIP-3 (Fig. 4) to induce conformational change and promote its oligomerization and formation of membrane pores [94]. The ability of TNF to induce two forms of cell death by its action highlights the importance of this pathway in I/R-induced inflammatory processes [95, 96].

A different pathway is mediated by TRAF2 which recruits two anti-apoptotic proteins cellular inhibitor of apoptosis protein 1 (cIAP-1) and 2 (cIAP-2) with E3 ligase activity [89]. Self-ubiquitination of cIAP-1/2 allows the recruitment of the linear ubiquitin chain assembly complex (LUBAC) (Fig. 4) to ubiquitinate several proteins, including NF-κB essential modulator (NEMO), or RIP-1 which promotes its kinase activity and induces TAB2/TAK1/IKKβ/NF-κB inflammatory pathway [16, 93]. Similarity between RIP-1 signaling and TLR signaling pathways shows that miRNAs targeting TLR pathway would be effective inhibitors of TNF-induced RIP-1 inflammatory signaling as well. Induction of TNF signaling resulting in the activation of NF-κB stimulates mTOR activation which in turn enhances NF-κB activity [93, 97]. This process can be inhibited by miR-24 via decreasing nuclear translocation and DNA binding of NF-κB as was shown in vascular smooth muscle cells (VSMCs) [98]. The presented molecular mechanism represents an intersection of TNF and mTOR signaling pathways, therefore miRNAs targeting the mTOR pathway may also intercept the inflammatory signal [32, 80].

Mitogen-activated protein kinases (MAPKs) are thought to be also activated by TRAF2-mediated recruitment of E3 ligating enzymes [89]. Several MAPKs, like JNKs, or p38 are related to tissue damage and cytokine production during I/R injury [99]. Both JNKs and p38 can activate the transcription factor AP1 and induce the expression of TNF to promote inflammation in multiple tissues [100]. As was already mentioned, this process can be attenuated by miR-199a, as was shown in a transcriptomic study on smooth muscle cells with implications in I/R injury settings [44, 79].

Higher levels of serum TNF-α were associated with an increased expression of circulating isoform of miR-146a-5p and miR-155-5p and overexpression of these miRNAs was associated with endothelial dysfunction and impaired vessel relaxation [95, 101]. However, TNF-α may also regulate its effect during inflammation by the upregulation of anti-inflammatory miR-10a which targets both IRAK4 and TAK1 in synovial-like fibroblasts under inflammatory conditions [102]. These studies support the relationship between inflammatory miRNAs and a higher risk of cardiovascular diseases in chronic inflammatory states [103]. Functional endothelium is crucial for the uninterrupted blood flow. Pathological conditions affecting its function may cause the formation of thrombi and subsequent ischemic damage to tissues [5]. The miR-126 was shown to be a mediator of endothelial inflammation, proven to be constitutively expressed mainly in endothelial cells (ECs), in which it reduces TNF-α induced VCAM-1 expression [104]. Additional targets of miR-126 are negative regulators of VEGF signaling sprouty-related, EVH1 domain-containing protein 1 (SPRED1), and phosphoinositide-3-kinase regulatory subunit 2 (PIK3R2) which cause decreased tube formation and sprouting of ECs during angiogenesis, in addition to reduced cell migration and proliferation [105]. Furthermore, miR-126 might suppress inflammation and ROS production in ECs by modulation of HMGB1 expression [106]. These studies highlight that miRNAs can simultaneously modulate various pathological processes during I/R and improve tissue rescue prognosis if maintained at effective levels [3]. The TNF-α upregulates miR-17 and miR-31 in human umbilical vein endothelial cells (HUVECs) in which the miR-17 inhibits the ICAM-1 expression in a negative feedback loop while miR-31 downregulates eNOS pathway resulting in endothelial dysfunction [107, 108]. Several other miRNAs are upregulated by TNF-α as of some of the already described miRNAs like miR-21, miR-146a/b and miR-155 which mostly exert an anti-inflammatory effect [79, 109]. Additionally, miR-146 is a powerful positive regulator of neovascularization induced in hypoxic tissues and miR-21 targets PTEN, the inhibitor of VEGF-induced angiogenesis [110, 111]. The mentioned studies show that miRNAs precisely regulate the inflammatory TNF signaling and their dysregulation might potentially promote inadequate response of endothelium to TNF-α during I/R injury. In addition, the miR-181 was also studied in the context of inflamed endothelium. Expression of miR-181 suppresses endothelial activation by targeting importin-α3, a crucial protein for nuclear translocation of NF-κB, both in in vitro and in vivo models of vascular endothelium and negatively regulates the stability of TNF-α mRNA in macrophages [112, 113]. These findings suggest a therapeutic potential of this miRNA for inflamed endothelium and its protective function from the effects of I/R injury. Furthermore, miR-181 has a crucial role in the selection of CD4^+^CD8^+^ double-positive T cells and CD4^+^ cells in the thymus. The mechanism of regulation of the selection process is based on the interaction of miR-181a with the protein tyrosine phosphatase N22 (PTPN22) and dual-specificity phosphatases 5 and 6 (DUSP5/6) and SHP-2 [114]. Inhibition of these phosphatases leads to the deletion of autoreactive T-cell clones. The importance of this miRNA in clonal selection has been confirmed by experiments with depleted miR-181a, in which reactivity against self-antigens was increased indicating abundance in self-reactive T cells [115]. These findings further underscore the therapeutical potential of this miRNA in maintaining optimal immune responses to I/R injury.

Results of a study on intestinal macrophages of pediatric patients has reported that miR-124 causes decreased production of TNF-α by targeting STAT3 and moreover, lower levels of miR-124 have been already linked to neuronal inflammation during acute spinal cord injury and cerebral I/R injury [116–118]. These results indicate that miR-124 is implicated in the inflammatory response by targeting TNF signaling and may serve as a potential therapeutic avenue in cerebral I/R injury to mitigate excessive cell death and tissue damage. Another miRNA linked to neuronal inflammation is miR-128 which was found to increase the levels of TNF-α and promote neuronal apoptosis after stroke [119] Similarly, higher miR-19a-3p levels were associated with increased TNF-α production and enhanced cerebral I/R injury [120]. On the contrary, miR-18b, miR-22 and miR-34c-5p downregulate TNF-α levels and protect against cerebral I/R injury [121–123].

Targeting TNF signaling by miRNAs can also have detrimental effects. The TNFR2 receptor present in Tregs is a positive regulator of their proliferation and immunosuppressive effects [124]. Therefore, inhibition of TNFR2 signaling within Treg cells may exacerbate tissue responses to inflammation by decreased production of IL-10, transforming growth factor β (TGF-β) or weakened effector T cell suppression [125]. A study on the Tregs in the tumor environment found that miR-125b-5p targets TNFR2 and reduces Treg proliferation along with their immunosuppressive function, leading to enhanced anti-tumor immunity [126]. The importance of TNFR2 in I/R-induced inflammatory response was studied in a mouse model of hind-limb ischemia. Results of this study have shown that the deletion of TNFR2 significantly enhanced inflammatory response and decreased post hind-limb ischemia recovery [127]. Such observations support the pro-inflammatory role of miR-125b-5p, however, the same miRNA was found to effectively protect the myocardium of transgenic mice from I/R injury and reduce infarct size by targeting the TRAF6/NF-κB pathway [128]. The opposing biological effects of miR-125b-5p in the mentioned studies highlight its cell-specific effects. Beyond their role in suppressing immune responses, Tregs also contribute to tissue repair and regeneration, which further promotes significance of TNF receptor network [129]. Angiogenesis is an important process in the regeneration of tissues after ischemic injury [1]. A study on vascular endothelial cells examined the expression of TNFR2 and found that its expression in endothelial cells positively impacts angiogenesis, proliferation, survival and migration of endothelial cells, while TNFR1 caused endothelial cell apoptosis [130]. The protective effect of TNF signaling through TNFR2 in I/R injury was further underscored by the study on adult infarct myocardium reporting decreased infarct size in TNFR1 knockout mice models [131].

## Anti-inflammatory effect of SIRT1 activity and its regulation during I/R injury

**Fig. 5:**
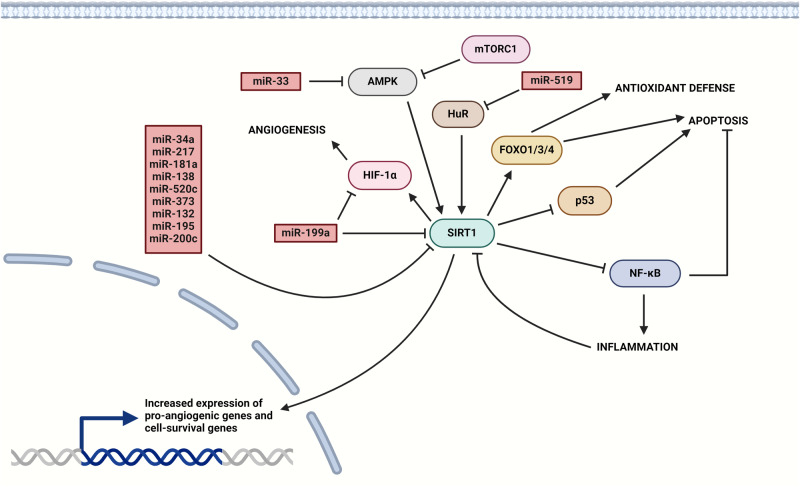
SIRT1 signaling involves multiple pathways. One of the pathways functions via deacetylation of p53 and NF-κB to suppress their activity and downstream pro-inflammatory and pro-apoptotic gene expression, resulting in dampened inflammation and increased cell survival. Furthermore, SIRT1 modulates activity of FOXO transcription factors and HIF-1α to promote cell proliferation, cell survival, ischemic tolerance, and antioxidant defenses. Negative regulation of SIRT1 is based on direct downregulation by several miRNAs or indirect downregulation by miRNAs targeting positive regulators of SIRT1, such as AMPK or HuR. Additionally, inflammatory response mediated by activated NF-κB and mTORC1 also inhibits SIRT1 signaling. (Created with BioRender.com).

The Sirtuin family of proteins belongs to class III histone deacetylases (HDAC III) and there are up to seven different sirtuins (SIRTs) in humans [132]. The first discovered member of the SIRT family was SIRT1 which is studied for its effects on longevity and regulation of biological processes like cellular senescence, apoptosis, oxidative stress, and inflammation [133]. Most of the pathophysiological processes connected to SIRT1 were related to its interaction with non-histone proteins (Fig. 5) including p53, forkhead box transcription factor 1/3/4 (FOXO 1/3/4), HIF-1α, and NF-κB to regulate the transcription of their target genes connected to apoptosis, antioxidant activity, angiogenesis, or inflammation [132].

Levels of SIRT1 are regulated by various feedback loops depending on the cellular state [134]. The transcriptional regulation of SIRT1 involves p53 downregulating SIRT1 expression, while FOXO3a or C-MYC, transcription factors influencing cell survival, upregulate it [26]. Crosstalk between SIRT1 and mTOR pathways is also a crucial regulatory pathway. Activation of mTORC1 along with its downstream effector ribosomal protein S6 kinase (S6K1) inhibits the activity of SIRT1 by phosphorylation, although SIRT1 activates S6K1 by deacetylation [135]. The main regulator of SIRT1 intracellular activity is the ratio of NAD^+^/NADH and changes in NAD^+^/NADH are related to the energetic state of cells [136]. Induction of DNA repair mechanisms includes the increased activity of poly-ADP ribose polymerase 1 (PARP1), an NAD^+^-dependent ribozyme that significantly depletes NAD^+^ levels and inhibits SIRT1 activity [137]. Oxidative stress during I/R injury may promote PI3K signaling which in turn upregulates miR-34a expression to downregulate SIRT1, potentially creating a positive feedback loop and promote manifestation of adverse effects of I/R injury [138]. An important sensor of the energetic state of cells is AMP-activated protein kinase (AMPK) [139]. It promotes SIRT1 deacetylase activity by increasing intracellular NAD^+^ levels and directly phosphorylates the enzyme [26]. In the study conducted by Ling et al. (2020), the inhibitory effect of mTORC1 on AMPK was explored, showing an additional negative regulatory mechanism of SIRT1 activity, particularly driven by inflammation-induced mTORC1 activity. Results of this study done on mammalian cultures like HEK293 cells or C2C12 myoblasts under nutrient stress conditions highlighted reciprocal regulation of mTORC1 and AMPK activities [140]. In the context of inflammation, mTORC1 induced by inflammatory signaling possibly decreases the anti-inflammatory potential of SIRT1 by inhibiting AMPK [139, 141]. This effect was observed in several studies using metformin – a potent AMPK regulator on both in vitro and in vivo models of various inflammatory conditions, including I/R injury. Relevant studies are well summarized in the review article by Du et al. (2022) [142].

The role of SIRT1 in maintaining optimal immune response is connected to the inhibition of NF-κB activity by deacetylation of its p65 subunit at lysine 310 residue and interestingly, inflammation also promotes the accumulation of SIRT1 at promoters of NF-κB targets to block their transcription [26, 143]. On the other hand, NF-κB signaling leads to the inhibition of SIRT1 which displays the antagonistic relationship between these two proteins [132]. The downregulation of SIRT1 mediated by miR-34a was studied in ECs and EPCs and showed that overexpression of miR-34a induced endothelial senescence by downregulating SIRT1 expression and additionally, the expression of miR-34a was found to be higher in older mice compared to younger ones, supporting results of studies confirming age-related SIRT1 decrease [144]. Additionally, miR-138, miR-181a, miR-199a and miR-217 downregulate SIRT1 (Fig. 5) as well [145–148]. In the study on human cardiomyocytes under oxidative stress simulating I/R injury, miR-138 was found to be upregulated and its levels correlated with decreased SIRT1 levels and increased rate of apoptosis [145]. Oxidative stress also induced miR-181a expression in astrocytes and microglia and enhanced neuroinflammation and neuronal apoptosis by downregulating SIRT1. These results suggest a potential role of miR-181a in cerebral I/R injury [146]. Age-related decrease of SIRT1 was studied on HUVECs and found that miR-217 promotes endothelial senescence by targeting SIRT1, resulting in inhibited proliferation, migration, angiogenesis and cell growth. This study partially explains the increased cardiovascular risk and poor tissue rescue prognosis observed in older patients suffering from I/R injury partly due to miRNA dysregulation [147]. The role of miR-199a in targeting SIRT1 was also studied in alveolar macrophages in mouse models of sepsis-induced lung injury. The increased rate of apoptosis observed in cells with upregulated miR-199a was alleviated by acute downregulation of this miRNA. These results highlight the potential of miRNA-based therapy in the treatment of severe inflammatory states like sepsis, observed also in patients with poor management of I/R injury [2, 3, 148]. Notably, miR-34a and miR-217 are upregulated in dysfunctional endothelium, demonstrating their role in the development of atherosclerosis due to senescent endothelium [149]. Upregulation of MMP-9 plays an important role in the rupture of atherosclerotic plaques and some miRNAs, such as miR-520c and miR-373, may target SIRT1 to stimulate MMP-9 expression [150]. Accumulation of lipids in non-adipose tissues results in lipid overload and cell death [151]. Furthermore, elevated production of ROS during reperfusion mainly affects lipids by peroxidation which produces lipid radicals such as hydroperoxides that are more stable molecules than ROS themselves [152]. Study on obesity-associated inflammation have confirmed the role of miR-132 in the production of pro-inflammatory cytokines by direct targeting of SIRT1 and therefore inhibiting SIRT1-mediated downregulation of NF-κB pathway resulting in the increased production of MCP-1 and IL-8 in primary human pre-adipocytes and in vitro differentiated adipocytes [153, 154]. Upregulation of miR-132 in HUVECs also promoted TNF-α induced apoptosis. The role of miR-195 was studied in the culture of cardiomyocytes from the neonatal mice and results have demonstrated that increased expression of miR-195 suppressed SIRT1 expression, enhanced ROS production, induced apoptosis and suppression of miR-195 had an opposite effect, interestingly even after palmitate-induced ROS generation, making the miR-195 a potential therapeutic target to alleviate myocardial lipotoxicity and resulting cardiac dysfunction, related to high levels of free fatty acids [153–156]. A study has reported that the binding of NF-κB to the promoter region of SIRT1 in VSMCs enhances its expression, however, the exact mechanism of NF-κB regulation of SIRT1 expression remains to be elucidated [26]. These findings potentially suggest that miRNAs targeting the activity of NF-κB, such as miR-181, miR-34a and others mentioned in previous sections, might affect SIRT1 levels without directly targeting its mRNA. If such effects were observed in further studies, then some of the anti-inflammatory miRNAs would also promote inflammation and ROS production in this manner.

Aging is associated with a decline in cardiovascular health and may be strongly affected by falling SIRT1 levels and ongoing chronic inflammation [157]. Studies have confirmed that SIRT1 levels are important for maintaining functional endothelial cells by promoting the production of NO by deacetylation and activation of endothelial nitric oxide synthase (eNOS) leading to vasodilation and vasorelaxation in the aortas [136]. Increased NO production by SIRT1 in vessels may be also attributed to its ability to induce soluble guanylyl cyclase in VSMCs [158]. Consistent decrease in SIRT1 levels during aging, therefore affects the responsiveness of the vasculature and promotes arterial stiffness [136]. Reportedly, miR-200c greatly impacts vascular function by directly targeting SIRT1, eNOS, and FOXO1, promoting cellular senescence, decreased NO production, increased ROS production, and decreased ROS scavenging capacity, therefore miR-200c represents an attractive therapeutical target in terms of I/R injury [159]. The 3-´UTR region of the SIRT1 transcript contains the binding site for HuR which can stabilize the bound transcript [26]. The level of HuR decreases substantially with age, thus may contribute to downregulation of SIRT1 [133]. A rise in oxidative stress during reperfusion may further decrease SIRT1 stability by activating checkpoint kinase 2 (Chk2), resulting in the phosphorylation of HuR and its subsequent dissociation from SIRT1 mRNA [26]. The RNA-stabilizing protein HuR is repressed by miR-519 (Fig. 5), which therefore indirectly regulates SIRT1 stability [160]. Inflammation during I/R upregulates miR-33 which targets AMPK, leading to a decrease in the intracellular availability of NAD^+^, thus negatively regulating SIRT1 activity [161–163]. Therefore, not only activity but also the transcription of SIRT1 is downregulated during I/R injury, explaining the impaired resolution of inflammation in damaged tissues of older patients [164]. Upregulation of miR-34a and reciprocal reduction of SIRT1 may also serve as potential biomarkers of the aging brain at a higher risk of negative outcomes of cerebral ischemia [141, 153].

The angiogenic potential of senescent endothelium is reduced, leading to the prolongation of the ischemic interval [28]. The miR-217 was found to be upregulated in old HUVECs, HAECs, and human coronary artery endothelial cells (HCAECs) and it was demonstrated that miR-217 modulates SIRT1/eNOS and SIRT1/FOXO1 axes to inhibit angiogenesis and promote atherosclerosis [153]. Changes in the expression of miR-199a in the model of cardiac ischemia have shown that hypoxia decreases miR-199a expression, causing an increase in SIRT1 and HIF-1α levels in cardiac myocytes which ameliorated cytotoxicity in the context of I/R injury [148]. However, SIRT1 might deacetylate HIF-1α which decreases its stability and function, suggesting a regulatory mechanism preventing chronic activation of HIF-1α signaling [165]. Such interaction may prove to be detrimental to the potential pharmacological therapy of I/R injury based on SIRT1 induction. Moreover, LPS-stimulated upregulation of miR-199a in alveolar macrophages enhanced the production of pro-inflammatory cytokines, hence the inhibition of miR-199a might provide a potential therapy for I/R injury, alleviating inflammation, stimulating angiogenesis, and increasing ischemic tolerance [153].

## Utilization of miRNAs as therapeutics

Effective treatment of I/R injury is currently mostly limited to controlling the ROS generation at the time of reperfusion [166]. The complexity of ROS-mediated signaling and cell death pathways, such as necroptosis, apoptosis or necrosis decreases the ability to precisely predict the tissue response to treatment [7]. Outcome of intervention is also dependent on the specific organ suffering from I/R injury [167].

Therapeutic potential of miRNAs has been proposed after the discovery of their ability to regulate gene expression [168]. Utilization of miRNAs in the potential therapy of I/R injury involves two strategies: miRNA replacement and miRNA inhibition therapy [3, 169]. MiRNA replacement therapy aims to establish physiological regulation of gene expression disrupted during I/R injury. This process uses miRNA mimics, synthetic analogs of pathologically downregulated miRNAs to replenish their levels [170]. On the other hand, miRNA inhibition silences or knocks down pathologically upregulated miRNAs that contribute to I/R pathology. This therapy is based on anti-miRs, which are single-stranded and chemically modified nucleic acids designed to bind and inhibit their miRNA targets [171]. Currently, bioinformatic predictions suggest that one miRNA molecule can regulate almost 200 distinct genes with plethora of different functions, which may enable them to simultaneously regulate multiple pathways but also complicates their translation to clinical practice [168]. Apart from vast array of potential off-target effects, their instability in bloodstream complicates efficient and targeted delivery, necessary for desired treatment outcome [172, 173]. Their presence in tissue fluids may additionally trigger innate immune responses mediated by TLR signaling, as was already mentioned [62]. Development of suitable delivery systems, such as lipid-based nanoparticles (LNPs) substantially enhances therapeutic potential of miRNAs by alleviating their immunogenicity, increasing their stability and providing a platform for targeted delivery [174]. Clinical efficiency of LNPs was already demonstrated by the utilization of LNP-encapsulated mRNA vaccines against COVID-19 [175]. However, complex regulatory networks of miRNAs combined with elusive changes in their expression pose a great challenge even nowadays [168]. Despite challenges, few of mentioned miRNAs such as miR-21, miR-155 or miR-34a have already been subjected to clinical trials which signifies their gradual establishment as therapeutic avenue [176]. The effects of all miRNAs mentioned in this review and their therapeutic potential in the context of I/R injury is summarized in Table 1. Rapid advances in nanotechnology, molecular biology and computational modelling drive the development of more efficient and safer miRNA-based therapies, which are yet to be verified in the settings of I/R injury.

**Table 1:** Effects of selected miRNAs and their therapeutic potential in the context of I/R injury.

miRNA	Molecular target	Experimental model	Expression	Biological consequence	Implication in I/R pathology	Therapeutic implication	Ref.
miR-99a	mTOR	Naive mouse CD4 + T cells undergoing differentiation	Upregulated	Increase in Treg cell population	Resolution of acute inflammation and prevention of further tissue damage or necrosis	miRNA replacement therapy	[38]
			Downregulated	Increase in Th17 cell population	Intense inflammatory reaction and poor tissue rescue prognosis		
miR-100	mTOR	Naive CD4 + T cells	Upregulated	Decrease in Treg cell population	Delayed resolution phase and overactive inflammatory response	miRNA inhibition therapy	[39]
			Downregulated	Increase in Treg cell population	Resolution of acute inflammation and prevention of further tissue damage or necrosis		
miR-451	Not particularly mentioned	Gastric cancer tissues and tumor-infiltrated T cells	Upregulated	Increased mTOR activity, Th17 cell population	Intense inflammatory reaction and poor tissue rescue prognosis	miRNA inhibition therapy	[40]
			Downregulated	Decreased mTOR activity and Th17 cell population	Resolution of acute inflammation and prevention of further tissue damage or necrosis		
miR-155	Not particularly mentioned	OT-I T cells	Upregulated	Increased effector response by CD8 T cells, decreased differentiation into central memory CD8 T cells	Enhanced intensity of inflammation and promotion of necrosis	miRNA inhibition therapy	[41]
			Downregulated	Weaker effector response and preferential differentiation into central memory CD8 T cells	Decreased severity of I/R injury and promotion of tissue regeneration, however potential robust inflammatory response to subsequent ischemic stimuli		
	MyD88	Human gastric cancer cell line AGS and HEK-293 cells	Upregulated	Attenuated inflammatory response	Decreased severity of I/R injury and faster resolution of inflammation	miRNA replacement therapy	[63]
			Downregulated	Enhanced inflammatory response	Increased severity of I/R injury and higher risk of systemic inflammation		
	TAB2	Human monocyte-derived dendritic cells	Upregulated	Decreased intensity of inflammatory response by TLR/IL-1 signaling	Decreased severity of I/R injury and attenuated inflammatory response	miRNA replacement therapy	[76]
			Downregulated	Increased intensity of inflammatory response by TLR/IL-1 signaling	Increased severity of I/R injury and poor tissue rescue prognosis		
miR-21	PTEN	Murine macrophage cell lines, primary macrophages and 4T1 Cells	Upregulated	Immune suppression, enhanced M2 transformation, increased VEGF expression and mTOR signaling	Improved resolution of inflammation and enhanced tissue regeneration	miRNA replacement therapy	[42]
			Downregulated	Enhanced M1 transformation and decreased mTOR signaling	Enhanced inflammatory response and slower tissue regeneration		
miR-1224-5	PLK1	Human normal osteoblast line and osteosarcome cell lines	Upregulated	Decreased proliferation, invasion, increased apoptosis and autophagic activity	Increased severity of I/R injury and poor tissue rescue prognosis	miRNA inhibition therapy	[43]
			Downregulated	Increased proliferation, invasion and reduced apoptosis	Decreased severity of I/R injury and enhanced regeneration of affected tissues		
miR-199a	mTOR	AC16 cardiomyocytes	Upregulated	Decreased mTOR signaling	Decreased severity of I/R injury and increased ischemic tolerance	miRNA replacement therapy	[44]
			Downregulated	Increased apoptosis and inhibited autophagy	Increased severity of I/R injury and decreased ischemic tolerance		
	SIRT1	Mouse alveolar macrophages	Upregulated	Increased inflammatory cytokine production and enhanced cellular apoptosis	Increased severity of I/R injury and poor tissue rescue prognosis with higher risk of systemic inflammation	miRNA inhibition therapy	[148]
			Downregulated	Inhibited production of inflammatory cytokines and inhibition of apoptotis	Decreased severity of I/R injury and improved tissue rescue prognosis with lower risk of systemic inflammation		
miR-223	NLRP3	H9c2 cardiomyocyte-like cells	Upregulated	Reduced cellular damage by oxidative stress	Decreased severity of I/R injury and improved tissue rescue prognosis	miRNA replacement therapy	[47]
			Downregulated	Decreased cellular viability in response to oxidative stress	Increased severity of I/R injury and higher risk of systemic inflammation		
	TLR4	Murine macrophages	Upregulated	Inhibited lipid deposition and inflammatory response	Decreased severity of I/R injury and further disease progression in patients with atherosclerosis	miRNA replacement therapy	[24]
			Downregulated	Enhanced inflammatory response and foam cell formation	Increased severity of I/R injury and further disease progression in patients with atherosclerosis		
miR-9	HuR	Human cardiomyocytes	Upregulated	Inhibited pyroptosis caused by hyperglycemia	Decreased severity of I/R injury due to unresolved inflammation and improved tissue rescue prognosis in diabetic patients	miRNA replacement therapy	[49]
			Downregulated	Enhanced activation of caspase-1 during hyperglycemia	Increased severity of I/R injury and higher risk of systemic inflammation in diabetic patients		
miR-30c-5p	FOXO3	Human aortic endothelial cells	Upregulated	Inhibition of pyroptosis caused by oxLDL	Decreased severity of I/R injury in patients with atherosclerosis	miRNA replacement therapy	[50]
			Downregulated	Activation of NLRP3 inflammasome by oxLDL	Increased severity of I/R injury in patients with atherosclerosis		
miR-30a	Keap1	Rheumatoid arthritis fibroblast-like synoviocytes	Upregulated	Activation of Nrf2 pathway and protection from oxidative stress	Decreased severity of I/R injury and improved tissue rescue prognosis	miRNA replacement therapy	[57]
			Downregulated	Severe oxidative stress injury	Increased severity of I/R injury and poor tissue rescue prognosis		
let-7i	TLR4	H69 human cholangiocytes	Upregulated	Attenuated immune response	Decreased severity of I/R injury and improved tissue rescue prognosis	miRNA replacement therapy	[62]
			Downregulated	Enhanced inflammatory response	Increased severity of I/R injury and higher risk of systemic inflammation		
miR-203-5p	MyD88	Macrophage RAW264.7 cells	Upregulated	Attenuated TLR signaling	Decreased severity of I/R injury and improved tissue rescue prognosis	miRNA replacement therapy	[64]
			Downregulated	Enhanced inflammatory signaling	Increased severity of I/R injury and higher risk of systemic inflammation		
miR-149-5p	MyD88	Macrophage RAW264.7 cells	Upregulated	Attenuated TLR signaling	Decreased severity of I/R injury and improved tissue rescue prognosis	miRNA replacement therapy	[65]
			Downregulated	Enhanced inflammatory signaling	Increased severity of I/R injury and higher risk of systemic inflammation		
miR-124-5p	MyD88	Macrophage RAW264.7 cells	Upregulated	Attenuated TLR signaling	Decreased severity of I/R injury and improved tissue rescue prognosis	miRNA replacement therapy	[66]
			Downregulated	Enhanced inflammatory signaling	Increased severity of I/R injury and higher risk of systemic inflammation		
miR-146a	IRAK1	THP-1 monocytes	Upregulated	Inhibited NF-κB signaling and pro-inflammatory cytokine production	Decreased severity of I/R and improved tissue rescue prognosis	miRNA replacement therapy	[67]
			Downregulated	Enhanced production of pro-inflammatory cytokines	Increased severity of I/R and poor tissue rescue prognosis		
	IRAK2	Human embryonic kidney HEK-293 cells	Upregulated	Attenuated inflammatory response to viral infection	Decreased severity of I/R injury, improved tissue rescue prognosis and lower risk of systemic inflammation	miRNA replacement therapy	[72]
			Downregulated	Enhanced production of type I interferons as a response to viral infection	Increased severity of I/R injury, poor tissue rescue prognosis and higher risk of systemic inflammation		
	TRAF6	THP-1, U937, HL-60, WEHI-3, BJAB and Mono-Mac-6 cell lines	Upregulated	Attenuation of TLR and cytokine signaling	Decreased severity of I/R and improved tissue rescue prognosis	miRNA replacement therapy	[68]
			Downregulated	Enhanced inflammatory signaling	Increased severity of I/R and poor tissue rescue prognosis		
miR-133-5p	IRAK1	Human embryonic kidney HEK-293T cells and primary coelomocytes	Upregulated	Attenuated inflammatory response and phagocytosis	Decreased severity of I/R injury and lower rate of foam cell formation in patients with atherosclerosis	miRNA replacement therapy	[73]
			Downregulated	Enhanced inflammatory response and phagocytosis	Increased severity of I/R injury and higher rate of foam cell formation in patients with atherosclerosis		
miR-142-5p	IRAK1	Macrophage RAW264.7 cells	Upregulated	Reduced production of pro-inflammatory mediators	Decreased severity of I/R injury and lower risk of systemic inflammation	miRNA replacement therapy	[74]
			Downregulated	Enhanced production of pro-inflammatory mediators	Increased severity of I/R injury and higher risk of systemic inflammation		
miR-93-5p	IRAK4	Macrophage RAW264.7 cells	Upregulated	Suppressed NF-κB signaling and pro-inflammatory cytokine production	Decreased severity of I/R injury and lower risk of systemic inflammation	miRNA replacement therapy	[75]
			Downregulated	Increased production of pro-inflammatory cytokines	Increased severity of I/R injury and higher risk of systemic inflammation		
miR-3178-5p	TRAF3	GES-1 Cells	Upregulated	Decreased activation of NF-κB signaling	Decreased severity of I/R injury and improved tissue rescue prognosis	miRNA replacement therapy	[82]
			Downregulated	Increased production of pro-inflammatory cytokines	Increased severity of I/R injury and higher risk of systemic inflammation		
miR-3473-5p	TRAF3	Macrophage RAW264.7 cells and TC-1 cell line	Upregulated	Decreased production of TNF-α, reduced rate of apoptosis and increased survival	Decreased severity of I/R injury, improved tissue rescue prognosis and lower risk of systemic inflammation	miRNA replacement therapy	[83]
			Downregulated	Increased production of TNF-α, increased rate of apoptosis and reduced survival	Increased severity of I/R injury, poor tissue rescue prognosis and higher risk of systemic inflammation		
miR-155-5p	SIRT1	Human umbilical vein endothelial cells	Upregulated	Endothelial senescence and apoptosis induced by TNF-α	Increased severity of I/R injury, poor tissue rescue prognosis and higher risk of systemic inflammation	miRNA inhibition therapy	[101]
			Downregulated	Enhanced cell proliferation and reduced TNF-α-induced senescence	Decreased severity of I/R injury, improved tissue rescue prognosis and lower risk of systemic inflammation		
miR-24	HMGB1	Vascular smooth muscle cells	Upregulated	Reduced cell proliferation, migration, NF-κB nuclear translocation and cytokine production during high-glucose treamtment	Decreased severity of I/R injury and attenuated inflammatory response in diabetic patients	miRNA replacement therapy	[98]
			Downregulated	Increased cell proliferation, migration and inflammation during high-glucose treatment	Increased severity of I/R injury and enhanced inflammatory response in diabetic patients		
miR-199a-5p	ASK1	Human embryonic kidney HEK293 cells and primary cultures of human bladder UE cells	Upregulated	Reduced impact of TNF signaling in hypoxia	Decreased severity of I/R injury and increased ischemic tolerance	miRNA replacement therapy	[79]
			Downregulated	Lower bladder contractility and enhanced inflammatory response during hypoxia	Increased severity of I/R injury and poor tissue rescue prognosis		
	IKKβ	Human embryonic kidney HEK293 cells and primary cultures of human bladder UE cells	Upregulated	Reduced impact of TNF signaling and nuclear translocation of NF-κB during hypoxia	Decreased severity of I/R injury and increased ischemic tolerance		
			Downregulated	Lower bladder contractility and enhanced inflammatory response during hypoxia	Increased severity of I/R injury and poor tissue rescue prognosis		
miR-10a	IRAK4	Rheumatoid arthritis fibroblast-like synoviocytes	Upregulated	Attenuated NF-κB activation, suppressed inflammatory cytokine production and lower rate of cell proliferation under inflammatory conditions	Decreased severity of I/R injury, improved tissue rescue prognosis and lower risk of systemic inflammation	miRNA replacement therapy	[102]
			Downregulated	Accelerated NF-κB activation, enhanced expression of inflammatory cytokines and increased cell proliferation under inflammatory conditions	Increased severity of I/R injury, poor tissue rescue prognosis and higher risk of systemic inflammation		
	TAK1	Rheumatoid arthritis fibroblast-like synoviocytes	Upregulated	Attenuated NF-κB activation, suppressed inflammatory cytokine production and lower rate of cell proliferation under inflammatory conditions	Decreased severity of I/R injury, improved tissue rescue prognosis and lower risk of systemic inflammation	miRNA replacement therapy	
			Downregulated	Accelerated NF-κB activation, enhanced expression of inflammatory cytokines and increased cell proliferation under inflammatory conditions	Increased severity of I/R injury, poor tissue rescue prognosis and higher risk of systemic inflammation		
miR-126	VCAM-1	Human umbilical vein endothelial cells	Upregulated	Decreased leukocyte adherence to endothelial cells under inflammatory conditions	Decreased severity of I/R injury, attenuated inflammation and improved tissue rescue prognosis	miRNA replacement therapy	[104]
			Downregulated	Increased leukocyte adherence to endothelial cells under inflammatory conditions	Increased severity of I/R injury, robust inflammatory response and poor tissue rescue prognosis		
	SPRED1	Macrophage RAW264.7 cells and human dermal microvascular endothelial cell line HMEC-1	Upregulated	Enhanced angiogenesis during ischemic conditions	Decreased severity of I/R injury, increased ischemic tolerance and improved tissue rescue prognosis	miRNA replacement therapy	[105]
			Downregulated	Slower rate of angiogenesis in ischemic conditions	Increased severity of I/R injury, reduced ischemic tolerance and poor tissue rescue prognosis		
	PIK3R2	Macrophage RAW264.7 cells and human dermal microvascular endothelial cell line HMEC-1	Upregulated	Enhanced angiogenesis during ischemic conditions	Decreased severity of I/R injury, increased ischemic tolerance and improved tissue rescue prognosis	miRNA replacement therapy	
			Downregulated	Slower rate of angiogenesis in ischemic conditions	Increased severity of I/R injury, reduced ischemic tolerance and poor tissue rescue prognosis		
	HMGB1	Human umbilical vein endothelial cells	Upregulated	Attenuated inflammation and oxidative stress under hyperglycemic condition	Decreased severity of I/R injury and improved tissue rescue prognosis	miRNA replacement therapy	[106]
			Downregulated	Enhanced inflammation and oxidative stress under hyperglycemic condition	Increased severity of I/R injury and poor tissue rescue prognosis		
miR-17	ICAM-1	Human umbilical vein endothelial cells and human dermal fibroblasts	Upregulated	Decreased neutrophil adhesion to endothelial cells in inflammatory conditions	Decreased severity of I/R injury, attenuated inflammation and improved tissue rescue prognosis	miRNA replacement therapy	[107]
			Downregulated	Increased neutrophil adhesion to endothelial cells in inflammatory conditions	Increased severity of I/R injury, robust inflammatory response and poor tissue rescue prognosis		
miR-31	eNOS	Human umbilical vein endothelial cells and cultured human placental arterial vessels	Upregulated	Induced endothelial dysfunction, vascular remodelling in response to TNF-α treatment	Increased severity of I/R injury and poor tissue rescue prognosis	miRNA inhibition therapy	[108]
			Downregulated	Reduced negative effect of TNF-α on endothelial cells	Decreased severity of I/R injuryand improved tissue rescue prognosis		
miR-181	importin α3	Human umbilical vein endothelial cells	Upregulated	Reduced downstream NF-κB signaling and leukocyte influx under inflammatory conditions	Decreased severity of I/R injury and lower risk of systemic inflammation	miRNA replacement therapy	[112]
			Downregulated	Enhanced inflammatory signaling and cytokine production	Increased severity of I/R injury and higher risk of systemic inflammation		
	TNF-α	THP-1 monocytes and type II lung epithelial cells A549	Upregulated	Decreased TNF-α levels and immunoparalysis in sepsis	Decreased severity of I/R injury and lower risk of systemic inflammation but poor survival prognosis in septic state	miRNA replacement therapy	[113]
			Downregulated	Increased TNF-α, GM-CSF and INF-γ levels in sepsis	Increased severity of I/R injury and higher risk of systemic inflammation but improved survival prognosis in septic state		
miR-124	STAT3	Macrophage RAW264.7 cells and THP-1 monocytes	Upregulated	Attenuated production of IL-6 and TNF-α and inhibited macrophage activation in response to inflammatory stimuli	Decreased severity of I/R injury and improved tissue rescue prognosis	miRNA replacement therapy	[116]
			Downregulated	Enhanced production of IL-6 and TNF-α leading to further macrophage activation in response to inflammatory stimuli	Increased severity of I/R injury a poor tissue rescue prognosis		
miR-128	CREB1	Mouse neurons and astrocytes	Upregulated	Inhibited CREB1 and BDNF expression leading to enhanced apoptosis during oxygen-glucose deprivation	Increased severity of I/R injury and decreased ischemic tolerance leading to poor tissue rescue prognosis	miRNA inhibition therapy	[119]
			Downregulated	Facilitated proliferation of cells and inhibited apoptosis during oxygen-glucose deprivation	Decreased severity of I/R injury and increased ischemic tolerance leading to improved tissue rescue prognosis		
miR-19a-3p	IGFBP3	Neuroblastoma SH-SY5Y cells	Upregulated	Decreased cell viablity, enhanced inflammation and apoptosis during oxygen-glucose deprivation	Increased severity of I/R injury and decreased ischemic tolerance leading to poor tissue rescue prognosis	miRNA inhibition therapy	[120]
			Downregulated	Increased cell viability, inhibited inflammation and apoptosis during oxygen-glucose deprivation	Decreased severity of I/R injury and increased ischemic tolerance leading to improved tissue rescue prognosis		
miR-18b	ANX3A	Neuroblastoma SH-SY5Y cells	Upregulated	Increased cell viability, inhibited inflammation and apoptosis during oxygen-glucose deprivation	Decreased severity of I/R injury and increased ischemic tolerance leading to improved tissue rescue prognosis	miRNA replacement therapy	[122]
			Downregulated	Decreased cell viablity, enhanced inflammation and apoptosis during oxygen-glucose deprivation	Increased severity of I/R injury and decreased ischemic tolerance leading to poor tissue rescue prognosis		
miR-22	Not particularly mentioned	Rat pheochromocytoma cells line PC12	Upregulated	Downregulation of inflammatory factors, and suppresion of the MIP-2, PGE2, COX-2 and iNOS expression during oxygen-glucose deprivation	Decreased severity of I/R injury and attenuated inflammatory response leading to improved tissue rescue prognosis	miRNA replacement therapy	[121]
			Downregulated	Induced protein expression of NF-κB, p38 and MAPK during oxygen-glucose deprivation	Increased severity of I/R injury and enhanced inflammatory response leading to poor tissue rescue prognosis		
miR-34c-5p	NCOA1	Primary cortical neuron cell culture	Upregulated	Inhibited activity of NF-κB, decreased levels of inflammatory cytokines and increased Bcl-2 expression during oxygen-glucose deprivation	Decreased severity of I/R injury and attenuated inflammatory response leading to improved tissue rescue prognosis	miRNA replacement therapy	[123]
			Downregulated	Increased levels of inflammatory cytokines and neuronal apoptosis during oxygen-glucose deprivation	Increased severity of I/R injury and enhanced inflammatory response leading to poor tissue rescue prognosis		
miR-34a	SIRT1	MCF-7, A549, MDA-MB-231, SKOV3, HCT-116, HCT116p53_/_ cell lines	Upregulated	Suppression of SIRT1 and Nrf2 pathways leading to increased cancer cell death due to oxidative stress	Increased severity of I/R injury caused by decreased resistance to oxidative stress leading to poor tissue rescue prognosis	miRNA inhibition therapy	[138]
			Downregulated	Active SIRT1 and Nrf2 pathways causing increased resistance to oxidative stress in cancer cells	Decreased severity of I/R injury caused by increased resistance to oxidative stress leading to improved tissue rescue prognosis		
miR-138	SIRT1	Human cardiomyocyte cell lines AC16, HCM, HCFB, and CCC-HEH-2	Upregulated	Dowregulation of SIRT1 and upregulation p53 signaling inducing apoptosis under oxidative stress	Increased severity of I/R injury caused by decreased resistance to oxidative stress leading to poor tissue rescue prognosis	miRNA inhibition therapy	[145]
			Downregulated	Elevated SIRT1 levels leading to increased oxidative stress resistance and inhibited apoptosis under oxidative stress	Decreased severity of I/R injury caused by increased resistance to oxidative stress leading to improved tissue rescue prognosis		
miR-181a	SIRT1	Male Sprague-Dawley rats	Upregulated	Increased neuronal apoptosis, astrocyte and microglia activation, neuroinflammation and oxidative stress in immature epileptic rats	Increased severity of I/R injury caused by decreased resistance to oxidative stress leading to poor tissue rescue prognosis	miRNA inhibition therapy	[146]
			Downregulated	Inhibited neuronal apoptosis, astrocyte and microglia activation, neuroinflammation, oxidative stress and decreased cognitive dysfunction	Decreased severity of I/R injury caused by increased resistance to oxidative stress leading to improved tissue rescue prognosis		
miR-217	SIRT1	Human umbilical vascular endothelial cells	Upregulated	Inhibited proliferation, migration and angiogenesis to promote endothelial senescence	Increased severity of I/R injury and poor tissue rescue prognosis due to inhibited angiogenesis	miRNA inhibition therapy	[147]
			Downregulated	Optimal proliferation, migration and angiogenesis during endothelial cells growth	Decreased severity of I/R injury and improved tissue rescue prognosis by promoting angiogenesis		
miR-520c	SIRT1	Human fibrosarcoma HT1080 cells	Upregulated	Increase in MMP-9 expression, activation of NF-κB signaling and enhanced cell migration	Increased severity of I/R injury, poor tissue rescue prognosis and higher risk of atherosclerotic plaque rupture	miRNA inhibition therapy	[150]
			Downregulated	Decrease in MMP-9 levels, inhibition of NF-κB signaling and suppressed cell migration	Decreased severity of I/R injury, improved tissue rescue prognosis and lower risk of atherosclerotic plaque rupture		
miR-373	SIRT1	Human fibrosarcoma HT1080 cells	Upregulated	Increase in MMP-9 expression, activation of NF-κB signaling and enhanced cell migration	Increased severity of I/R injury, poor tissue rescue prognosis and higher risk of atherosclerotic plaque rupture	miRNA inhibition therapy	
			Downregulated	Decrease in MMP-9 levels, inhibition of NF-κB signaling and suppressed cell migration	Decreased severity of I/R injury, improved tissue rescue prognosis and lower risk of atherosclerotic plaque rupture		
miR-132	SIRT1	Human umbilical vein endothelial cells	Upregulated	Promotion of pro-inflammatory processes related to enhanced lipogenesis and cholesterogenesis, increased apoptosis and inhibited proliferation and migration under inflammatory conditions	Increased severity of I/R injury and poor tissue rescue prognosis in patients with atherosclerosis	miRNA inhibition therapy	[153]
			Downregulated	Suppressed pro-inflammatory processes and apoptosis related to lipid metabolism and increased proliferation and migration under inflammatory conditions	Decreased severity of I/R injury and improved tissue rescue prognosis in patients with atherosclerosis		
miR-195	SIRT1	Neonatal mouse cardiomyocyte culture	Upregulated	Increased ROS production and apoptosis induction after palmitate treatment	Increased severity of I/R injury and poor tissue rescue prognons in patients with atherogenic lipid profile	miRNA inhibition therapy	[154]
			Downregulated	Decreased ROS production and inhibited apoptosis after palmitate treatment	Decreased severity of I/R injury and improved tissue rescue prognons in patients with atherogenic lipid profile		
miR-200c	SIRT1	Human umbilical vein endothelial cells	Upregulated	Enhanced ROS production and apoptosis and reduction in the expression of FOXO1 and ROS scavengers under oxidative stress	Increased severity of I/R injury caused by decreased resistance to oxidative stress leading to poor tissue rescue prognosis	miRNA inhibition therapy	[157]
			Downregulated	Decrease in ROS production and apoptosis and increased expression of FOXO1 and ROS scavengers	Decreased severity of I/R injury caused by increased resistance to oxidative stress leading to improved tissue rescue prognosis		
	eNOS	Human umbilical vein endothelial cells	Upregulated	Decrease in nitric oxide production and SIRT1 levels	Increased severity of I/R injury caused by decreased resistance to oxidative stress leading to poor tissue rescue prognosis	miRNA inhibition therapy	
			Downregulated	Increase in nitric oxide production and SIRT1 levels	Decreased severity of I/R injury caused by increased resistance to oxidative stress leading to improved tissue rescue prognosis		
	FOXO1	Human umbilical vein endothelial cells	Upregulated	Decrease in ROS scavengers production and increase in ROS production under oxidative stress	Increased severity of I/R injury caused by decreased resistance to oxidative stress leading to poor tissue rescue prognosis	miRNA inhibition therapy	
			Downregulated	Increase in the ROS scavenger levels and reduced ROS production	Decreased severity of I/R injury caused by increased resistance to oxidative stress leading to improved tissue rescue prognosis		
miR-519	HuR	human malignant meningioma cell line IOMM-Lee cells and benign grade 1 meningioma cell line Ben-Men-1 cells	Upregulated	Reduced cell proliferation, migration and overall tumor suppressive effect	Increased severity of I/R injury and poor tissue rescue prognosis	miRNA inhibition therapy	[158]
			Downregulated	Increased cell proliferation, migration and tumorigenic effect	Decreased severity of I/R injury and improved tissue rescue prognosis		
miR-33	AMPK	HepG2, Huh7 and COS-7 cell lines	Upregulated	Reduced ketogenesis and fatty acid oxidation, increased intracellular cholesterol levels	Increased severity of I/R injury and poor tissue rescue prognosis due to lipotoxicity-induced cell death	miRNA inhibition therapy	[161]
			Downregulated	Increased ketogenesis and fatty acid oxidation, reduced intracellular cholesterol levels	Decreased severity of I/R injury and improved tissue rescue prognosis due to boosted intracellular lipid metabolism		
miR-125-5p	TNFR2	Tregs in tumor environment	Upregulated	Increased immune response towards tumor tissue	Enhanced inflammatory response and slower tissue regeneration after I/R injury	miRNA inhibition therapy	[126]
			Downregulated	Enhanced proliferation and migration of tumor cells	Decreased severity of I/R injury and improved tissue rescue prognosis		
	TRAF6	Mouse myocardial cells	Upregulated	Reduced infarct size, attenuated inflammatory response and reduced rate of apoptosis	Decreased severity of I/R injury caused by increased resistance to oxidative stress leading to improved tissue rescue prognosis	miRNA replacement therapy	[128]
			Downregulated	Enhanced rate of apoptosis and reduced tolerance towards oxidative stress	Increased severity of I/R injury caused by decreased resistance to oxidative stress leading to poor tissue rescue prognosis		

## Conclusion

Ischemia/reperfusion injury represents a serious complication in ischemic diseases of the cardiovascular system. Understanding the role of miRNAs in the immune responses during I/R injury is of great importance as it provides insights into the regulatory mechanisms underlying the inflammatory processes and potential therapeutic targets for intervention. The current body of research describes the ability of miRNAs to regulate key processes and signaling cascades, including NLRP3 inflammasome, TLR signaling, mTOR pathway, TNF signaling and SIRT1 activity, all of which modulate inflammation and cellular responses to oxidative stress caused by I/R injury. Overall, deciphering the intricate interplay between cell- and tissue-specific expression of miRNAs and the immune response in ischemia/reperfusion injury holds promise for uncovering novel diagnostic markers and therapeutic strategies for managing this complex condition.

## Data Availability

Data sharing is not applicable to this article as no datasets were generated or analyzed during the current study.
